# AI-guided virtual biopsy: Automated differentiation of cerebral gliomas from other benign and malignant MRI findings using deep learning

**DOI:** 10.1093/noajnl/vdae225

**Published:** 2025-01-20

**Authors:** Mathias Holtkamp, Vicky Parmar, René Hosch, Luca Salhöfer, Hanna Styczen, Yan Li, Marcel Opitz, Martin Glas, Nika Guberina, Karsten Wrede, Cornelius Deuschl, Michael Forsting, Felix Nensa, Lale Umutlu, Johannes Haubold

**Affiliations:** Institute for Artificial Intelligence in Medicine, University Hospital Essen, Germany; Institute of Diagnostic and Interventional Radiology and Neuroradiology, University Hospital Essen, Essen, Germany; Institute for Artificial Intelligence in Medicine, University Hospital Essen, Germany; Institute for Artificial Intelligence in Medicine, University Hospital Essen, Germany; Institute for Artificial Intelligence in Medicine, University Hospital Essen, Germany; Institute of Diagnostic and Interventional Radiology and Neuroradiology, University Hospital Essen, Essen, Germany; Institute of Diagnostic and Interventional Radiology and Neuroradiology, University Hospital Essen, Essen, Germany; Institute of Diagnostic and Interventional Radiology and Neuroradiology, University Hospital Essen, Essen, Germany; Institute of Diagnostic and Interventional Radiology and Neuroradiology, University Hospital Essen, Essen, Germany; Division of Clinical Neurooncology, Department of Neurology, University Hospital Essen, Essen, Germany; Department of Radiotherapy, University Hospital Essen, Germany; Department of Neurosurgery, University Hospital Essen, Essen, Germany; Institute of Diagnostic and Interventional Radiology and Neuroradiology, University Hospital Essen, Essen, Germany; Institute of Diagnostic and Interventional Radiology and Neuroradiology, University Hospital Essen, Essen, Germany; Institute for Artificial Intelligence in Medicine, University Hospital Essen, Germany; Institute of Diagnostic and Interventional Radiology and Neuroradiology, University Hospital Essen, Essen, Germany; Institute of Diagnostic and Interventional Radiology and Neuroradiology, University Hospital Essen, Essen, Germany; Institute for Artificial Intelligence in Medicine, University Hospital Essen, Germany; Institute of Diagnostic and Interventional Radiology and Neuroradiology, University Hospital Essen, Essen, Germany

**Keywords:** gliomas, machine learning, MRI analysis, pathology differentiation, virtual biopsy

## Abstract

**Background:**

This study aimed to develop an automated algorithm to noninvasively distinguish gliomas from other intracranial pathologies, preventing misdiagnosis and ensuring accurate analysis before further glioma assessment.

**Methods:**

A cohort of 1280 patients with a variety of intracranial pathologies was included. It comprised 218 gliomas (mean age 54.76 ± 13.74 years; 136 males, 82 females), 514 patients with brain metastases (mean age 59.28 ± 12.36 years; 228 males, 286 females), 366 patients with inflammatory lesions (mean age 41.94 ± 14.57 years; 142 males, 224 females), 99 intracerebral hemorrhages (mean age 62.68 ± 16.64 years; 56 males, 43 females), and 83 meningiomas (mean age 63.99 ± 13.31 years; 25 males, 58 females). Radiomic features were extracted from fluid-attenuated inversion recovery (FLAIR), contrast-enhanced, and noncontrast T1-weighted MR sequences. Subcohorts, with 80% for training and 20% for testing, were established for model validation. Machine learning models, primarily XGBoost, were trained to distinguish gliomas from other pathologies.

**Results:**

The study demonstrated promising results in distinguishing gliomas from various intracranial pathologies. The best-performing model consistently achieved high area-under-the-curve (AUC) values, indicating strong discriminatory power across multiple distinctions, including gliomas versus metastases (AUC = 0.96), gliomas versus inflammatory lesions (AUC = 1.0), gliomas versus intracerebral hemorrhages (AUC = 0.99), gliomas versus meningiomas (AUC = 0.98). Additionally, across all these entities, gliomas had an AUC of 0.94.

**Conclusions:**

The study presents an automated approach that effectively distinguishes gliomas from common intracranial pathologies. This can serve as a quality control upstream to further artificial-intelligence-based genetic analysis of cerebral gliomas.

**Key Points**

- Fully automated intracerebral pathology analysis enables the noninvasive differentiation of intracranial pathologies.
- Artificial intelligence models demonstrated strong diagnostic performance with area-under-the-curve values over 0.95.
- Provides a safety net for virtual glioma biopsies.

**Importance of the Study**

In this study, an automated algorithm was developed to noninvasively distinguish gliomas from other intracranial pathologies. Existing clinical algorithms often confuse gliomas with other pathologies. The new algorithm uses machine learning and radiomics to accurately differentiate gliomas from metastases, inflammatory lesions, intracerebral hemorrhages, and meningiomas, achieving an area under the curve of over 0.95. This high diagnostic accuracy serves as a safety net before further genetic analysis of gliomas, significantly improving diagnostic reliability. It reduces the risk of misdiagnosis and enhances patient safety. This automated differentiation is a crucial step toward a comprehensive virtual biopsy of brain tumors, contributing to more precise and individualized treatment planning.

Cerebral gliomas are primary brain tumors that can be categorized into numerous subtypes.^1^ The recent 2021 World Health Organization (WHO) Classification of Tumors of the Central Nervous System highlights the importance of molecular diagnostics in gliomas, a focus that was already highlighted in the 2016 WHO Classification.^2,3^ This requires invasive tissue sampling as a standard procedure. To acquire samples, a stereotactic needle biopsy is routinely performed. This is accompanied by complication rates of 3%–25% and a mortality rate of 0%–4%.^4–9^ To circumvent these side effects while still developing a therapeutic concept aligned with the molecular status, there is a significant demand for noninvasive tissue analysis.

In addition to conventional biopsy, there are novel approaches for determining the genetic profile of gliomas. In recent years, the significant potential of MRI-based image analyses for decoding gliomas has already been demonstrated.^10–16^ An important approach for extracting information from brain MR images is the utilization of feature-based radiomics and deep learning-based methods.^17–25^ Numerous research studies have already been conducted to predict specific mutations.^15,26–30^ Some studies have also analyzed the complete genetic profile.^10,31^ Here, for example, Haubold et al. predicted grading and genetic profiling using a fully automated radiomics analysis based on multiparametric MRI examinations. By analyzing the FLAIR sequence, as well as noncontrast and contrast-enhanced T1-weighted sequences, a robust automated prediction model was established. This model predicted the grading as well as isocitrate dehydrogenase 1 and 2 (IDH1/2) mutation, O6-methylguanine-DNA methyltransferase (MGMT) methylation status, chromosomal regions 1p and 19q (1p19q) co-deletion, and loss of ATRX expression in cerebral gliomas with an overall good performance.^31^ However, the presence of a glioma had to be confirmed in all studies. Until now, this confirmation has been obtained through invasive sample collection.

To avoid misclassifying other intracranial pathologies as gliomas without invasive biopsies and their potential side effects, we used the framework of Haubold et al.^31^ to develop a model capable of distinguishing between gliomas and other common intracranial pathologies.

The aim was to create an algorithm that, in routine clinical practice, classifies intracranial pathologies as either gliomas or non-gliomas, with the objective of automatically directing gliomas to the prediction model for genetic profiling.

## Methods

### Ethics Statement

This study adhered to all the guidelines prescribed by the institutional review board of the investigating hospital and was approved by the local ethics committee (Approval code: 21-10487-BO). The requirement for written informed consent was waived by the Institutional Review Board due to the retrospective design of the study. All data were completely anonymized prior to their use in the study.

### Study Cohort

MRIs of 1280 patients were enrolled, including 218 gliomas, 514 brain metastases, 366 inflammatory lesions (multiple sclerosis, acute disseminated encephalomyelitis, progressive multifocal leukoencephalopathy, and encephalitis), 99 intracerebral hemorrhages, and 83 meningiomas. To ensure data privacy and confidentiality, all patient data were rigorously anonymized before the integration of MRI data and clinical characteristics. The patients had a mean age of 56.26 ± 14.19 years (587 men [45.9%] and 693 women [54.1%]) (Table 1).

**Table 1.** Distribution of Age and Gender in the Cohort Across Various MRI Findings

	Age (mean ± SD years)	Gender (male/female)	Percentage (male/female)
Metastatic lesions	59.28 ± 12.36	228/286	44.35/55.65
Inflammatory lesions	41.94 ± 14.57	142/224	42.26/57.74
Intracranial hemorrhages	62.68 ± 16.64	56/43	56.57/43.43
Meningioma	63.99 ± 13.31	25/58	30.12/69.88
Gliomas	54.76 ± 13.74	136/82	62.35/37.65

### Magnetic Resonance Imaging

The MRI examinations were conducted at a single center, utilizing various 1.5 T (MAGNETOM Aera, MAGNETOM Avanto, MAGNETOM Espree, MAGNETOM Sonata, MAGNETOM Symphony) and 3 T (Biograph mMR, MAGNETOM Skyra, MAGNETOM Vida) MR machines from a single vendor (Siemens Healthineers). The study period spans from March 2002 to May 2023. For the radiomics analysis, the MR sequences, FLAIR, noncontrast, and contrast-enhanced T1-weighted sequences, were selected.

### Preprocessing

The initial step in the preprocessing phase involved resampling all 3 sequences, namely FLAIR, contrast-enhanced, and noncontrast-enhanced T1-weighted, to a uniform spatial resolution of (1., 1., 1.) mm^3^. This resampling procedure was executed using Advanced Normalization Tools (ANTs) in Python (ANTsPy),^32^ a Python package that encapsulates the functionalities of ANTs,^33^ a C++ biomedical image processing library, and harnesses the statistical capabilities of ANTsR.^34^ ANTsPy seamlessly integrates these tools with NumPy, scikit-learn, and the broader Python community.^32^

To ensure data anonymization and the removal of extracranial structures, a skull stripping was conducted. HD-BET,^35^ a publicly available algorithm renowned for its state-of-the-art performance, was employed for precise brain tissue extraction. Subsequently, to align all sequences within the same spatial orientation, coregistration was performed employing ANTsPy’s registration module.^36^ This process involved the rigid transformation technique, specifically a translation, to co-register FLAIR and contrast-enhanced T1-weighted sequences with noncontrast-enhanced T1-weighted images.

**Figure 1.**
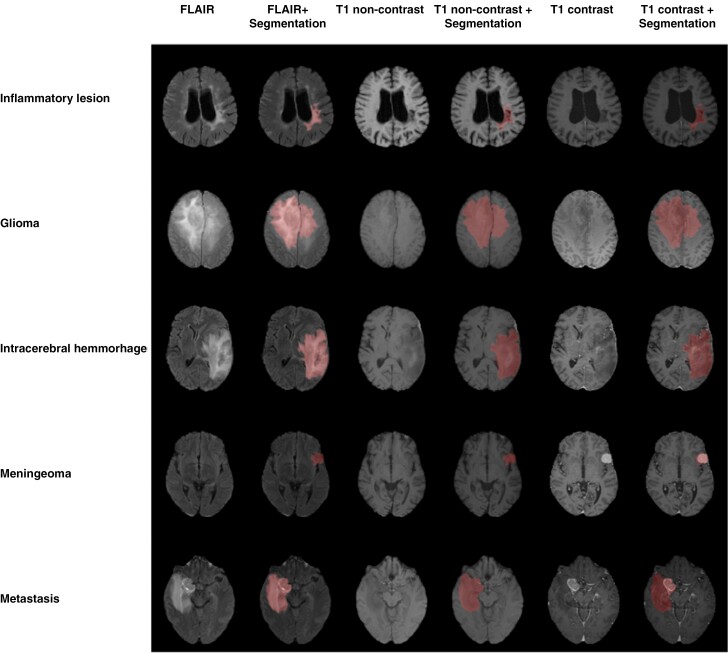
Examples of fully automated segmentations and their coregistration with the respective FLAIR, noncontrast T1-weighted sequence, and contrast-enhanced T1-weighted sequence.

The coregistered images were then utilized to generate automatic tumor segmentations (Figure 1) using HD-GLIO,^24,37^ an open-source algorithm that employs a nnU-Net architecture.^38^ HD-GLIO was trained using FLAIR, contrast-enhanced T1-weighted, noncontrast-enhanced T1-weighted, and T2-weighted sequences, although it’s noteworthy that the study cohort lacked T2-weighted sequences, necessitating the use of FLAIR as a surrogate for segmentation purposes which was validated with manual segmentations of cerebral gliomas by Haubold et al.^31^

### Feature Extraction

Subsequent to the generation of segmentations, the PyRadiomics software^39,40^ was employed to derive radiomic features from the segmented regions. The extracted feature set encompassed a comprehensive array of descriptors, including first-order statistical attributes, geometric features based on shape analysis, characteristics derived from Gray Level Co-Occurrence Matrix (GLCM), features based on Gray Level Run Length Matrix, attributes derived from Gray Level Size Zone Matrix, Neighboring Gray Tone Difference Matrix–related features, and features stemming from Gray Level Dependence Matrix analysis. Pertinent characteristics were derived from images subjected to diverse filter-based transformations, encompassing the Wavelet transformation, Laplacian of Gaussian (LoG) transformation, Local Binary Pattern 3D (LBP3D) transformation, and Gradient transformation.

### Train Test Split

In accordance with their respective medical conditions, the MR examinations of the 1280 patients were distributed across various subgroups, as described above. Subsequently, discrete subcohorts were established for model training and evaluation. Notably, all subcohorts adhered to a consistent stratification scheme, employing an 80% train and 20% test-splitting approach. Furthermore, these train and test splits were stratified to ensure the maintenance of a balanced ratio between positive and negative cases within both subsets.

This study delineated 5 discrete subcohorts. The distribution of both positive and negative cases within each of these subcohorts is detailed in Table 2 for reference.

**Table 2.** Distribution of Positive and Negative Cases in Different Subcohorts.

Cohort description	Train (positive/negative)	Test (positive/negative)
Gliomas vs. all other entities	174/849	44/213
Gliomas vs. metastatic lesions	174/411	44/103
Gliomas vs. meningioma	174/66	44/17
Gliomas vs. intracranial bleeding	174/79	44/20
Gliomas vs. inflammatory lesions	174/293	44/73

### Feature Selection

To mitigate noise stemming from the presence of redundant or closely correlated features, the BorutaPy,^41^ an implementation of the Boruta algorithm^42^ in the Python programming language, was employed for feature selection. As a method for selecting all the important features, it aims to cover all the key details related to a specific outcome. It’s worth mentioning that methods using groups of decision trees, like Random Forest, Gradient Boosted Trees, and Extra Trees Classifiers, are good at figuring out complex, non-straightforward relationships between factors, especially when there are not many data points compared to the number of factors (a situation called “small n, significant p”).^41^ XGBoost algorithm^43^ was specified as the estimator utilized within the BorutaPy framework to optimize the resultant feature set.

### Parameter Optimization and Model Evaluation

The tuning of XGBoost parameters was executed through the utilization of the Tree-structured Parzen Estimator sampler, integrated within the Optuna framework.^44,45^ Each optimization process encompassed a series of 100 iterations wherein parameters were stochastically sampled from a predefined parameter space. Within each iteration of the optimization procedure, a 10-fold cross-validation strategy was implemented, aiming to maximize the f1-score concerning the held-out fold from the cross-validation. Every subcohort underwent an identical optimization procedure.

During the training phase, we improved the models using the f1-score, which was chosen to balance precision and recall. The f1-score measures these two essential components of performance. When deciding on the final models for each classification assignment, we prioritized the area under the curve (AUC) of the receiver-operating characteristic (ROC) curve. AUC was chosen because it provides a more comprehensive perspective of the model’s discriminating power across all decision thresholds, making it a reliable evaluation indicator for ultimate model performance.

Table 3 presents the optimized parameters specific to each subcohort. To mitigate the risk of data leakage, hyperparameter tuning was exclusively conducted on the training dataset.

**Table 3.** Optimal Hyperparameters for Each Subcohort Selected Through Optuna

Parameter	Glioma vs. all other Pathologies	Glioma vs. metastasis	Glioma vs. inflammatory lesions	Glioma vs. intracerebral hemorrhage	Glioma vs. meningioma
booster	gbtree	gbtree	gbtree	dart	gbtree
grow_policy	depthwise	lossguide	depthwise	lossguide	depthwise
n_estimators	100	100	100	100	100
scale_pos_weight	4.885057	2.362069	1.683908	0.454023	0.37931
gamma	0	0.319064	0	0.001264	0
max_depth	6	3	6	5	6
lambda	1	0.043813	1	0.953963	1
alpha	0	0.398513	0	0.708249	0
eta	0.3	0.119043	0.3	0.691486	0.3
sample_type	uniform	uniform	uniform	uniform	uniform
normalize_type	tree	tree	tree	forest	tree
rate_drop	0	0	0	0.312374	0
skip_drop	0	0	0	0.988103	0

All models were trained with the objective function *binary:logistic and a random_state* of *42.*

For feature selection, BorutaPy incorporates a parameter denoted as “perc,” which governs the number of features to be selected. Lower values of “perc” result in the inclusion of a greater number of false positives as relevant features, albeit at the expense of omitting some genuinely pertinent features. To identify the most advantageous feature subset, various values of this parameter were systematically tested. Following the feature selection phase, hyperparameters for the XGBoost algorithm underwent optimization for each distinct feature set. Subsequently, an individual XGBoost model was trained for each subcohort, utilizing the hyperparameters ascertained through the hyperparameter tuning process and the specific features selected. Among the ensemble of models, the one that attained the highest AUC on the test set was designated as the definitive model.

### Baseline Evaluation With a Dummy Classifier

To benchmark the performance of our models, we employed a dummy classifier as a baseline. This classifier provides a reference point by generating predictions without utilizing any learned patterns from the data. Specifically, it operates using a “stratified” strategy, which accounts for class imbalance by generating predictions proportional to the class distribution within the dataset. This ensures that the predictions reflect the inherent imbalance in the data, rather than assuming uniform class probabilities.

The dummy classifier’s performance was evaluated using the same metrics as the primary models, including the AUC.

### Human Reader Evaluation

To provide a benchmark for human performance, 2 experienced neuroradiologists, each with more than 10 years of professional experience, independently evaluated half of the test dataset. Their assessments were conducted under the same conditions as the neural network to ensure a fair comparison. They were provided with only the 3 MRI sequences—FLAIR, noncontrast-enhanced T1-weighted, and contrast-enhanced T1-weighted—without access to additional clinical information or other imaging sequences.

### Preparation of the Manuscript

For linguistic assistance in composing the manuscript, ChatGPT (Version GPT-4.0), developed by OpenAI, was employed.

## Results

In total, a good performance was achieved in discriminating between gliomas and various subgroups of other intracranial pathologies. In the construction of the models, various combinations of algorithms for feature selection and hyperparameter optimization were employed. The best models for each classification task were selected based on the hyperparameter performance using Optuna. These models were then evaluated on the hidden test set, which was exclusively used for the final assessment. Corresponding accuracy, sensitivity, specificity, AUC values, and precision are presented in Table 4.

**Table 4.** Machine Learning Models, Number of Selected Features, and Performance Metrics (Area Under the Curve [AUC], Balanced Accuracy, F1 Score, Precision, Sensitivity, Specificity)

	Glioma vs. all other pathologies	Glioma vs. metastasis	Glioma vs. inflammatory lesions	Glioma vs. intracerebral hemorrhage	Glioma vs. meningioma
Base	XGB	RF	XGB	RF	XGB
AUC	0.94	0.96	1.0	0.99	0.98
Balanced accuracy	0.82	0.83	0.97	0.98	0.88
F1 score	0.71	0.77	0.96	0.98	0.96
Precision	0.75	0.82	0.94	1.0	0.92
Recall/sensitivity	0.68	0.73	0.98	0.96	1.0
Specificity	0.95	0.93	0.96	1.0	0.77
No of features	47	33	48	22	56

To provide a benchmark for model performance, the dummy classifier was evaluated using the same dataset. The AUC values for the dummy classifier were consistently near random chance, with values such as 0.45, 0.50, 0.51, 0.55, and 0.62.

In the context of distinguishing between gliomas and the other selected intracranial pathologies, consistently good results were achieved with AUC values >0.9.

**Figure 2.**
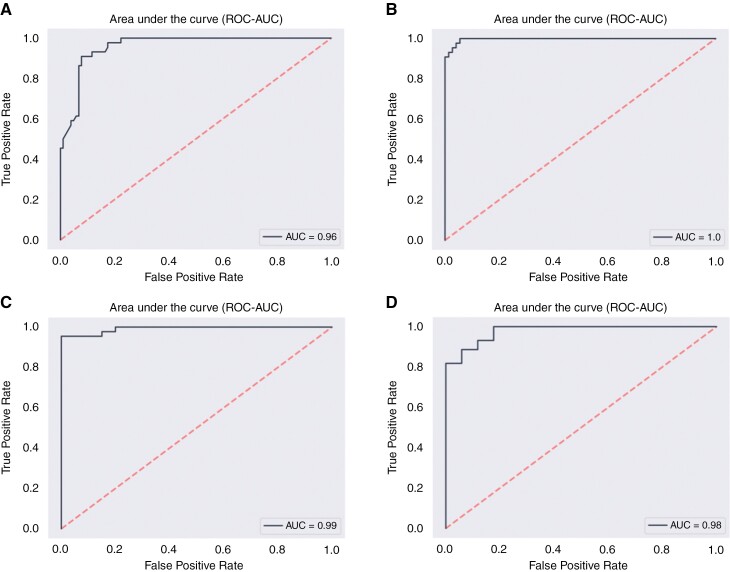
Receiver-operating curves (ROC) curves for predictive models discriminating gliomas from metastases (A), inflammatory lesions (B), intracerebral hemorrhages (C), and meningiomas (D).

The network was particularly good at differentiating between gliomas and metastases and had a high AUC of 0.96 (sensitivity 0.73, specificity 0.93). This predictive model was constructed using 33 selected features through feature selection. The ROC curve for these predictions is illustrated in Figure 2A.

The network designed for the differentiation of gliomas from inflammatory lesions yielded excellent results with a very good AUC of 1.0 (sensitivity 0.98, specificity 0.96) and contains 48 different features in its predictive model. Figure 2B shows the ROC curve for this model.

Furthermore, the network distinguishing gliomas from intracerebral hemorrhages achieved a very good AUC of 0.99 (sensitivity 0.96, specificity 1.0). For this model, 22 features were selected. The ROC curve is shown in Figure 2C.

The network for differentiating between gliomas and meningiomas delivered strong results. The predictive model obtained an AUC of 0.98 (sensitivity 1.0, specificity 0.77), employing a total of 56 selected features. Figure 2D presents the ROC curve for this predictive model.

**Figure 3.**
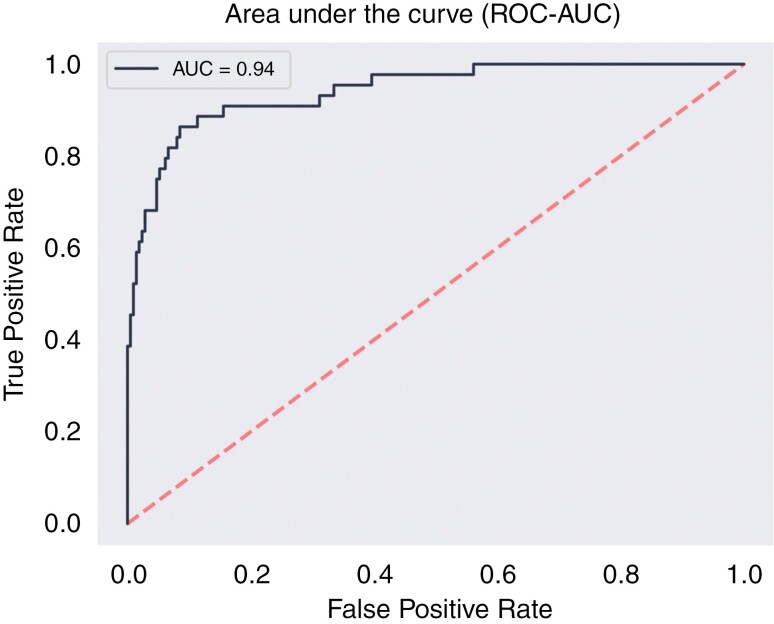
Receiver-operating curves (ROC) curves for predictive models discriminating gliomas from all other pathologies (metastases, inflammatory lesions, intracerebral hemorrhages, and meningiomas).

In the context of distinguishing gliomas from a combined group of intracranial pathologies, which include metastases, inflammatory lesions, intracerebral hemorrhages, and meningiomas, our neural network achieved good results. The model designed for this distinction achieved an AUC of 0.94 (sensitivity 0.68, specificity 0.95) utilizing a set of 30 selected features determined through a feature selection process. Figure 3 shows the ROC curve.

Additionally, 2 neuroradiologists with more than 10 years of professional experience achieved excellent results in distinguishing gliomas from the combined group of intracranial pathologies. Their performance included a sensitivity of 0.77 with a specificity of 0.99, and a sensitivity of 0.91 with a specificity of 0.97, respectively.

## Discussion

Our study focuses on differentiating gliomas from other common intracranial pathologies by analyzing radiomic features from routine cranial MRI scans in a fully automated pipeline. It therefore introduces an important safety net for algorithms with a focus on virtual biopsy of cerebral gliomas.

The results of our models demonstrated excellent performance in distinguishing gliomas from other intracranial pathologies. All models achieved an AUC of at least 0.96 (gliomas vs. metastases [AUC 0.96], vs. inflammatory lesions [AUC 1.0], vs. intracerebral hemorrhages [AUC 0.99], vs. meningiomas [AUC 0.98]). These differentiation outcomes align with existing literature, where previous studies have consistently demonstrated the capacity to effectively differentiate between various entities using MRI.^46–51^ For instance, Tsolaki et al. achieved high classification performance in the automatic differentiation of glioblastomas and metastases based on 3T MR spectroscopy and perfusion data.^48^ As early as 2009, initial studies involving perfusion maps and manual region of interest measurements revealed the capability to differentiate various cerebral pathologies based on image features. This study by Zacharaki et al. demonstrated the feasibility of distinguishing between different types of intracranial tumors. They successfully differentiated between metastases, meningiomas, gliomas, and glioblastomas in a relatively small cohort of 102 patients, achieving a model sensitivity range of 85%–87%. Although our models exhibit higher sensitivity and are based on a more extensive dataset, this early work by Zacharaki et al. highlighted the potential of machine learning for such differentiation tasks. Despite this potential, the low accuracy, the need for manual segmentation, and the complexity of imaging protocols have, to this day, hindered the integration of these approaches into clinical practice.^46^

In addition to comparing gliomas with individual intracranial pathologies, we also trained a prediction model to differentiate between gliomas and a combined group of intracranial pathologies, including metastases, inflammatory lesions, intracerebral hemorrhages, and meningiomas. While the individual models were highly accurate in distinguishing specific pathologies (eg, gliomas vs. metastases, gliomas vs. meningiomas), their use necessitates prior knowledge of the type of lesion being analyzed, which conflicts with the goal of a fully automated, biopsy-independent diagnostic process. In the scenario, to differentiate between gliomas and a combined group of intracranial pathologies, our neural network achieved noteworthy results with an AUC of 0.94. This performance demonstrates the combined model’s closer alignment with real-world clinical circumstances, where the underlying pathology is frequently unknown before further study, making it a more practical and usable approach for noninvasive diagnosis. However, it’s worth noting that the AUC in this context was slightly lower compared to the AUCs for distinguishing gliomas from individual intracranial pathologies. This could be due to the fact that the group with which gliomas are compared is very heterogeneous, which makes the differentiation more difficult. For an adequate safety net, however, it is crucial to differentiate gliomas from the most common pathologies and not a single pathology.

The results of our study, alongside prior research, highlight the performance of both humans and AI in this context. Rauschecker et al. (2020) reported that an AI system for MRI-based diagnosis achieved an accuracy of 91%, comparable to the 86% sensitivity of academic neuroradiologists, while significantly outperforming less specialized radiologists (radiology residents 56%, general radiologists 57%). In our study, the 2 experienced neuroradiologists demonstrated sensitivities of 77% and 91% in distinguishing gliomas from the combined group of other pathologies. By comparison, our algorithm achieved a sensitivity of 68% for the same task. However, specificity is of particular importance in our study, as the primary goal was to develop an algorithm capable of preventing non-glioma intracranial pathologies from being incorrectly routed for virtual biopsy evaluation. In this regard, our algorithm showed promising results, achieving a specificity of 95%, which was comparable to the neuroradiologists’ performances of 99% and 97%. These findings underscore the algorithm’s reliability in minimizing false positives and its potential as a safety control mechanism in clinical practice.

Studies on the differentiation of cerebral lesions often require complex MRI protocols, such as perfusion imaging.^46–48^ Additionally, some studies are typically conducted on specific MRI machines or limited to particular field strengths,^46–48,51^ which can introduce constraints that yield promising results but raise concerns about the generalizability of the approach. To circumvent these limitations, our objective was to adopt an approach with broad generalizability. For this purpose, we employed the prediction model by Haubold et al.,^31^ which makes predictions based on 3, in-brain imaging nearly universally applied MRI sequences: FLAIR, noncontrast-enhanced T1-weighted, and contrast-enhanced T1-weighted sequences. Furthermore, akin to Haubold et al.,^31^ we used a diverse set of MRI scanners operating at 1.5 and 3 Tesla to differentiate gliomas from other intracranial pathologies.

Another limitation in other studies aiming to distinguish intracranial pathologies is the manual or semi-automated segmentation method, which may introduce biases due to human influence and hinder clinical implementation due to the complexity of manual segmentations. To mitigate these potential biases, we employed an automated tumor segmentation using HD-GLIO.^13,14^ HD-GLIO is an algorithm utilizing a nnU-Net architecture^15^ trained on FLAIR, contrast-enhanced T1-weighted, noncontrast-enhanced T1-weighted, and T2-weighted sequences. Notably, Haubold et al. have previously demonstrated that in the case of the segmentation of cerebral gliomas, the network achieves a high segmentation efficiency without a T2-weighted sequence (DICE score of 0.81 ± 0.13).^31^ In this context, however, we have not explicitly shown in the present study that other pathologies are well segmented by this network. This is because with this study we wanted to place a control functionality in front of the virtual biopsy of cerebral gliomas so that other pathologies are not incorrectly classified into genetic profiles of cerebral gliomas. If separate dedicated segmentation networks were used, a pooled comparison that most closely matches this functionality would not be adequately possible. The use of separate segmentation networks would also contradict the initial situation that the pathology is unknown.

Overall, our study successfully achieved its primary objective of developing and evaluating a noninvasive AI-based model for distinguishing gliomas from other prevalent intracranial pathologies. The consistently high AUC values attained by the models for differentiation between gliomas and other common intracranial pathologies underscore the fulfillment of our primary research goal. The inclusion of a diverse and extensive dataset ensures that our findings possess a high degree of generalizability, rendering them relevant for a broad clinical context. The utilization of universally applicable MRI sequences and the incorporation of automated tumor segmentation to mitigate human-induced biases collectively enhance the study’s contributions.

Nevertheless, despite the promising results, our study is not without limitations. First, this study employed a retrospective and single-center approach. Further validation of these findings should involve a prospective multicenter study. While several key differential diagnoses for gliomas were examined in this work, there exist other intracranial pathologies for which differentiation models should be developed in future research. However, it is important to note that the pathologies chosen for this study are among the most common, which means that the current non-inclusion of rarer pathologies, due to their lower incidence, also leads to a relatively small number of misdiagnoses.

Although MRIs from different 1.5 and 3 Tesla MRI scanners were included in our study for generalizability, it is important to point out that these scanners were all from a single manufacturer, which could potentially bias the results. However, it is important to emphasize that our study included a very large cohort of 1280 patients, which included a variety of MRI protocols. The size of the patient group and the variety of MRI techniques employed add to the robustness of our findings.

## Conclusions

In summary, our study demonstrated a versatile solution for a noninvasive fully automated AI-based differentiation of cerebral gliomas from other intracranial pathologies. It shows a possible approach for the introduction of control functionalities in the analysis of the genetic profile of cerebral gliomas. The introduction of such control functionalities is an important next step before the clinical implementation of a virtual biopsy of cerebral gliomas.

## Data Availability

The data supporting the findings of this study will be made available upon reasonable request.
